# An Account of Diseases in an Eastern District of London, from Nov. 20 to Dec. 20, 1810

**Published:** 1811-01

**Authors:** 


### *An Account of Diseases in an Eastern District of London, from Nov. 20 to Dec. 20, 1810.*

| ACUTE DISEASES. | |
|---|---|
| Perepneumonia Notha | 2 |
| Cynanche Tonsillaris | 2 |
| Rubeola | 5 |
| Variola | 4 |
| Rheumatismus Acutus | 1 |
| **CHRONIC DISEASES.** | |
| Tussis | 10 |
| Dyspnœa | 7 |
| Tussis cum Dyspnœa | 8 |
| Catarrhus | 5 |
| Raucedo | 2 |
| Hœmoptysis | 4 |
| Phthisis Pulmonalis | 3 |
| Ophthalmia | 2 |
| Dyspepsia | 3 |
| Gastrodynia | 2 |
| Diarrhæa | 5 |
| Hæmorrhois | 3 |
| Dysuria | 4 |
| Vertigo | 4 |
| Rheumatismus Chronicus | 16 |
| **PUERPERAL DISEASES.** | |
| Ephemera | 6 |
| Menorrhagia Lochialis | 5 |
| Mastodynia | 4 |
| Rhagas Papillæ | 2 |
| Stranguria | 2 |
| **INFANTILE DISEASES.** | |
| Erysipelas Infantile | 4 |
| Aphthæ | 5 |
| Convulsio | 3 |
| Herpes | 6 |

Having now made some advances in the winter quarter of the year, we may expect that those diseases which are peculiar to this season, will make their appearance. The weather has hitherto, indeed, been very mild, so that the diseases referred to have not appeared in their most alarming form.

The immense quantity of rain which has lately fallen has been productive of an uncommon humidity of the atmosphere, which has rendered coughs and catarrhs very frequent; but these have not been attended with those symptoms of inflammation which usually prevail under the influence of the north and north-easterly winds. Rheumatism, of the chronic species, has very frequently occurred. In some constitutions this disease is particularly troublesome during a warm and moist state of the atmosphere. The heat has afforded relief to some patients, and cold has generally increased their pain; in other cases just the reverse of this has been the consequence. This very common complaint is, in some cases, relieved by an addition of warm clothing, and by the increased temperature of a warm bed, but in others it is very much aggravated by these very circumstances. Instances are not unfrequent where persons subject to rheumatic affections, after sitting near a fire for some time, or even occupying any part of a heated room, feel a pain in their joints, particularly in the knees, accompanied with a restlessness which is more distressing than the pain itself, and which is most speedily relieved by a removal into a cool room.

